# A retrospective study of reproductive outcomes after fertility-sparing surgery and postoperative adjuvant chemotherapy in malignant ovarian germ cell tumors and sex cord-stromal tumors

**DOI:** 10.1186/s13048-017-0348-x

**Published:** 2017-07-27

**Authors:** Ning Zhang, Ruifang Chen, Keqin Hua, Ying Zhang

**Affiliations:** Department of Gynecology, The Obstetrics and Gynecology Hospital of Fudan University, 128 Shenyang RD, Shanghai, 200090 China

**Keywords:** Malignant germ cell tumors, Malignant sex cord-stromal tumors, Fertility-sparing surgery, Postoperative adjuvant chemotherapy, Reproductive outcomes

## Abstract

**Background:**

To retrospectively investigate reproductive outcomes after fertility-sparing surgery and postoperative adjuvant chemotherapy in malignant ovarian germ cell tumors (MOGCT) and sex cord-stromal tumors (SCST).

**Methods:**

Data from 32 MOGCT (6 dysgerminomas, 6 yolk sac tumors, 17 immature teratomas, and 3 mixed germ cell tumors) and 9 SCST (4 granulosa cell tumors and 5 sertoli-leydig cell tumors) aged from 18 to 35, treated in the Obstetrics and Gynecology Hospital of Fudan University from October 2003 to October 2013 were collected and analyzed.

**Results:**

Average follow up was (86.3 ± 34.4) months. Average diagnosed age was (25.3 ± 3.5) years. Average chemotherapy course was (4.4 ± 1.3) times. Patients are all living. Thirty one patients (75.6%) reported normal menstrual cycles. Twelve patients (29.3%) wished to conceive, 10 (83.3%) naturally conceived and 8 (66.7%) had lived birth.

**Conclusions:**

Reproductive outcomes after fertility-sparing surgery and postoperative adjuvant chemotherapy in MOGCT and SCST are favorable, meanwhile education and consultation is needed.

## Background

Malignant ovarian germ cell tumors (MOGCT) and sex cord-stromal tumors (SCST) are not common. MOGCTs arise from primordial germ cells. Dysgerminoma is the most common histology of MOGCT, followed by immature teratoma and endodermal sinus tumor. SCSTs develop from the gonadal non-germ-cell components [1], including granulosa cell tumors, granulosa-theca tumors, and Sertoli-Leydig cell tumors. MOGCT and SCST are generally occurred in young women, considered low-grade, sensitive to chemotherapy, and good prognosis [2–4]. The 5-year survival rate is about 85% [5] in MOGCT, and 97% [6] in SCST. Fertility preservation treatment can be achieved in most cases.

According to American National Comprehensive Cancer Network (NCCN) 2017 guideline for ovarian cancer, the standard treatment regimen for MOGCT and stage IA/IC SCST with fertility desiring is fertility-sparing surgery. That is preservation of the uterus and the contralateral ovary with staging procedure. For younger patients with early-stage diseases, comprehensive staging can be omitted. After surgery, adjuvant chemotherapy of bleomycin, etoposide, and cisplatin (BEP) or platinum-based thermotherapy will recommend to certain patients.

However, pregnancy rate and live birth outcomes after such treatment are not well established of these two diseases. We composed this retrospective study to report and analyze the reproductive outcomes of MOGCT and SCST patients with fertility preservation in our hospital.

## Methods

Thirty two MOGCT and 9 SCST patients conducted fertility-sparing surgery and adjuvant chemotherapy with complete follow up data, younger than 35 years old at first treatment, continuously disease-free with minimum follow-up of 2 years in the Obstetrics and Gynecology Hospital of Fudan University from October 2003 to October 2013 were retrospectively investigated.

Diagnosis of MOGCT or SCST was revealed by pelvic mass, abdominal pain, growing ascites, and arising of tumor markers like α-fetoprotein (AFP), human choionic gonadotophin (HCG) and inhibin. The diagnosis was confirmed by surgical procedure and pathological diagnosis. Pathological diagnosis was confirmed according to the 2014 World Health Organization classification of tumors of female reproductive organs by expert gynecological pathologists in our hospital. Tumors were staged according to the International Federation of Gynecology and Obstetrics (FIGO) 2000 staging system for ovarian cancer.

Clinical characteristics including patient age, clinical stage, operation type, chemotherapy regimen, chemotherapy courses, survival length and follow up time were collected and recorded. Reproductive outcomes including pregnancy rate and live birth rate were investigated. Follow-up data until January 31, 2017 was collected from hospital records and telephone interview. This study was approved by the Ethics Committee of the Obstetrics and Gynecology Hospital of Fudan University. Oral informed consents were obtained from all patients.

Patients of MOGCT who desire fertility preservation can be treated with fertility-sparing surgery regardless of stages. SCST patients of stage IA or IC desire fertility preservation can be treated with fertility-sparing treatment. Fertility preservation operation means preserve uterus and the contralateral ovary with or without comprehensive staging (omentectomy and lymphadenectomy) by open laparotomy or laparoscopy. Gynecological oncologists participated in all the operations. For adolescent patients of early-stages, comprehensive staging was omitted.

Chemotherapy was scheduled for embryonal tumors, endodermal sinus tumors, stage II to IV dysgerminoma, stage I, grade 2 to 3, or stage II to IV immature teratoma. Stage I SCST with high-risk factors, such as tumor rupture, stage IC, tumor size >10-15 cm, poorly differentiated were also recommended chemotherapy. Chemotherapy schemes included BEP, BVP (bleomycin/vincristine/platinum) and VAC (vincristine/actinomycin/cyclophosphamide). In this study, all patients received chemotherapy, but with different schemes and courses.

Statistical analysis was performed using SPSS version 19.0 software (SPSS Inc., Chicago, IL). Continuous variables were described as mean and standard deviation. Student’s t test and nonparametric tests were used as appropriate. Chi-squared test and Fisher’s exact test were used for group comparisons for categorical variables. The correlation was assessed with Spearman correlation coefficients. Differences were considered to be significant at *p* < 0.05.

## Results

There were 45 patients underwent fertility-sparing surgery and postoperative adjuvant chemotherapy in the Obstetrics and Gynecology Hospital of Fudan University from October 2003 to October 2013. Among them 41 patients had completed follow up data. The missed follow-up rate was 8.9%. Clinical characteristics of the 41 patients are revealed in Table 1. Average patients’ age was (25.3 ± 3.5) years old, range from 18 to 31. Average follow-up duration was (86.3 ± 34.4) months, range from 39 to 159 months. Among all 41 followed up patients, there were 32 germ cell tumors: 6 dysgerminomas, 6 yolk sac tumors, 3 mixed germ cell tumors and 17 immature teratomas, among those there were 2 cases of grade 3, 9 cases of grade 2 and 6 cases of grade 1. Among 9 sex cord-stromal tumors, there were 4 granulosa cell tumors and 5 sertoli-leydig cell tumors. Thirty eight patients were diagnosed in FIGO stage I, 2 in stage II and 1 patient with immature teratoma was stage III. All the tumors in 41 patients were unilateral.

**Table 1:** Clinical characteristics of malignant germ cell tumor and sex cord-stromal tumor

Characteristic	Germ cell tumor	Sex cord-stromal tumor	*P* value
	(n = 32)	(n = 9)	
Follow up (months)	85.8±36.0	87.7±30.2	0.889
Average diagnosed age (years old)	25.2±3.5	25.8±3.8	0.644
Clinical stage			
I	29	9	/
II	2	0	/
III	1	0	/
Operation			
Laparoscopic/abdominal	5/27	4/5	0.087
Complete staging/not staging	14/18	3/6	0.711
Chemotherapy			
BEP	27	7	
PVB	4	2	
VAC	1	0	
Chemotherapy Courses	4.4±1.3	4.2±1.2	0.708
GnRHa injection	14/32	1/9	0.119
Normal menstruation	24/32	7/9	0.864
Pregnancy/willing to conceive	7/9	1/3	/
Live birth/willing to conceive	6/9	1/3	/
Abortion (unexpected pregnancy)	0	2	/
Completion surgery after childbearing	0	0	/

*BEP* bleomycin/etoposide/platinum, *BVP* bleomycin/vincristine/platinum, *VAC* vincristine/actinomycin/cyclophosphamide, *GnRHa* gonadotropin-releasing hormone agonist

Fertility-sparing surgery with completed surgical staging including pelvic lymphadenectomy and omentectomy was performed in 17 patients. Other patients underwent unilateral salpingo-oophorectomy (USO). No severe complication was recorded in this study. Thirty four patients undertook BEP chemotherapy, 6 with BVP and 1 with VAC. Chemotherapy courses differ from 2 to 7, with the average of (4.4 ± 1.3) courses.

Forty one patients are all living. Thirty one patients (75.6%) reported normal menstrual cycles, while others complained of light volume or shortened duration of menstruation. Menstruation status had no relationship with patients’ age (*rs* = 0.195, *P* = 0.227), clinical stage (*rs* = 0.209, *P* = 0.195), operation type (*rs* = −0.263, *P* = 0.101), nor chemotherapy courses (*rs* = −0.014, *P* = 0.932).

In all 41 patients, 12 patients (29.3%) wished to conceive, others neither not married, nor do not willing to. 8(66.7%) patients naturally conceived and lived birth without critical birth defects. There were 2 abortions because of unwilling pregnancy. The pregnant patients all conceived naturally and the pregnancy occurred during 1 to 4 years after treatment, no typical assisted reproductive technology was used (Table 2). In our study, no patient completed staging surgery after childbearing.

**Table 2:** Characteristics of 8 naturally conceived and lived birth patients

	Age of initial Diagnosis	Diagnosis	Stage	Diagnosis year	Operation	Operation type	Chemo therapy	Course	GnRHa injection	Interval of pregnancy and treatment
1	30	Dysgerminoma	Ia	May 2007	USO	Laparotomy	BEP	4	N	2 year
2	30	Yolk sac	Ic	May 2009	USO Completed staging	Laparotomy	BEP	6	N	2 year
3	25	Immature teratoma	Ia	Jun 2010	USO	Laparotomy	BEP	2	N	4 year
4	21	Immature teratoma	Ia	Mar 2004	USO	Laparotomy	VAC	4	N	2 year
5	24	Immature teratoma	IIIb	Jul 2006	USO Completed staging	Laparotomy	BEP	6	N	3 year
6	24	Immature teratoma	Ia	Mar 2004	USO	Laparotomy	BVP	4	N	1 year
7	25	Dysgerminoma	Ic	Jan 2013	USO Completed staging	Laparotomy	BEP	4	Y	2 year
8	27	Sertoli-leydig	Ia	Jan 2008	USO	Laparotomy	BEP	3	N	3 year

*USO* unilateral salpingo-oophorectomy, *BEP* bleomycin/etoposide/platinum, *BVP* bleomycin/vincristine/platinum, *VAC* vincristine/actinomycin/cyclophosphamide, *GnRHa* gonadotropin-releasing hormone agonist

## Discussion

The prognosis of MOGCT and SCST is much better than epithelial ovarian cancer. Patients who underwent fertility-sparing surgery were able to maintain ovarian function and fertility after tumor therapy [7]. From our results, 75.6% of the patients had regular menstruation after surgery and chemotherapy. In our hospital, laparoscopic surgery was comprehensively developed around 2010. The majority of patients in this study were treated by laparotomy and most of pregnant patients were in laparotomy group. We do not regularly biopsy the normal ovary as it could produce adhesion and scar, which could possibly cause infertility. And second-look surgery is not regularly conducted in our hospital.

Cancer survivors are always worried about the risks of cancer recurrence and the severe adverse impacts on their offspring. Compared to sibling controls, elective abortion rate among female cancer survivors was much higher [8]. In our study, 2 abortions were recorded because of unwilling conceive. Studies revealed that specific effects on pregnancy from chemotherapy without radiotherapy were generally few [9]. Female cancer survivors treated with chemotherapy and/or radiotherapy during childhood do not have higher risks of chromosomal syndromes, single gene disorders, or congenital anomalies in their children [10–14]. In our study, no severe congenital anomaly was reported, but we need long-time follow up.

The optimal time of pregnancy after cancer treatment is not verified. As the time period of follicle development is six months, it is prudent to wait that time period after chemotherapy [15]. And we also recommend a comprehensive assessment of cancer alleviation or cure before attempting conception. In our study, all the pregnancies occurred during 1 to 4 years after treatment. And all pregnant patients conceived naturally without assisted reproductive technology. We suggest fertility preservation before cancer treatment or as early as possible so as to maximize the reproductive potential of all cancer survivors [16]. And for some patients, certain assisted reproductive technology may be helpful.

Education and consultation regarding future reproductive plan is very important for cancer patients and their families [17]. But only half of female cancer survivors received reproductive health counseling [18]. In this study, this data is in lack unfortunately.

From 2010 after, we began to recommend gonadotropin-releasing hormone agonists (GnRHa) for ovarian function protection during chemotherapy without randomization. The efficacy of GnRHa for prevention of ovarian toxicity is still controversial [19]. In this retrospective study, no specific benefit of GnRHa was revealed.

## Conclusions

Although fertility-sparing surgery and postoperative adjuvant chemotherapy was suitable and safety for MOGCT and SCST, reproductive outcomes are favorable, multiple center study should be conducted to verify our conclusion. Educational consultation about following up and reproductive health should be taken for all the patients and their families.

## Abbreviations

AFP: α-fetoprotein
BEP: Bleomycin, etoposide, and cisplatin
BVP: Bleomycin/vincristine/platinum
FIGO: International Federation of Gynecology and Obstetrics
GnRHa: Gonadotropin-releasing hormone agonists
HCG: Human choionic gonadotophin
MOGCT: Malignant ovarian germ cell tumors
NCCN: American National Comprehensive Cancer Network
SCST: Sex cord-stromal tumors
VAC: Vincristine/actinomycin/cyclophosphamide
